# Efficient Blood-Brain Barrier Opening in Primates with Neuronavigation-Guided Ultrasound and Real-Time Acoustic Mapping

**DOI:** 10.1038/s41598-018-25904-9

**Published:** 2018-05-22

**Authors:** Shih-Ying Wu, Christian Aurup, Carlos Sierra Sanchez, Julien Grondin, Wenlan Zheng, Hermes Kamimura, Vincent P. Ferrera, Elisa E. Konofagou

**Affiliations:** 1 Department of Biomedical Engineering, Columbia University, New York, New York, USA; 2 Department of Neuroscience, Columbia University, New York, New York, USA; 3 Department of Radiology, Columbia University, New York, New York, USA

## Abstract

Brain diseases including neurological disorders and tumors remain under treated due to the challenge to access the brain, and blood-brain barrier (BBB) restricting drug delivery which, also profoundly limits the development of pharmacological treatment. Focused ultrasound (FUS) with microbubbles is the sole method to open the BBB noninvasively, locally, and transiently and facilitate drug delivery, while translation to the clinic is challenging due to long procedure, targeting limitations, or invasiveness of current systems. In order to provide rapid, flexible yet precise applications, we have designed a noninvasive FUS and monitoring system with the protocol tested in monkeys (from in silico preplanning and simulation, real-time targeting and acoustic mapping, to post-treatment assessment). With a short procedure (30 min) similar to current clinical imaging duration or radiation therapy, the achieved targeting (both cerebral cortex and subcortical structures) and monitoring accuracy was close to the predicted 2-mm lower limit. This system would enable rapid clinical transcranial FUS applications outside of the MRI system without a stereotactic frame, thereby benefiting patients especially in the elderly population.

## Introduction

Despite the increasing need of efficacious brain treatments due to the continuous growth of world population and average age increase, brain diseases including neurological disorders and tumors remain poorly treated due to the challenge of access through the skull and the blood-brain barrier (BBB) for drug delivery^1^. Focused ultrasound (FUS) is the sole technique for treating the brain noninvasively and locally with ablation (thermal effects)^2,3^ and BBB opening for drug delivery (mechanical effects through cavitation)^3–8^. It is also a valuable tool to study brain function through neuromodulation^9,10^. With the announcement of US Food and Drug Administration approval to treat essential tremors with FUS ablation^11^, several clinical trials are underway worldwide^3,12^ including FUS ablation to treat Parkinson’s disease^13^, BBB opening for chemotherapy of glioblastoma^14^, and BBB opening for treating Alzheimer’s disease^15^.

The key to FUS treatment success is an efficient system that provides FUS sonication and monitoring. Moreover, rapid application is crucial to accommodate a broad and especially old patient population with minimal cost and easy re-application. Magnetic resonance-guided FUS (MRgFUS) system has been used to open the BBB and/or ablate brain tissue with temperature monitored by MRI^2,16^. However, this involves placement of the patient inside the MRI scanner, and the long procedure takes upwards of 3 h due to the long imaging times to confirm targeting^15,17^. This long procedure not only could be difficult for the elderly, the high cost also constraints the application of multiple treatments significantly. Furthermore, BBB opening is a mechanical effect that cannot be monitored with MRI. While another implantable ultrasound device needs invasive surgery without targeting flexibility and monitoring^14^. Therefore, a more flexible system is yet to be established for BBB opening.

The stereotactic FUS system has the advantages of providing precise targeting independent of the MRI system, and can be coupled with acoustic monitoring for BBB opening as previously shown in non-human primates^18–20^. This targeting method can be achieved by acquiring a preliminary scan (MRI, CT, etc.) with stereotaxis to serve as a personalized brain atlas^18,20^. While this frame-based method can be restricted by the stereotactic manipulator and varying accuracy due to the lack of feedback on the positioning, utilizing a frameless stereotaxis known as neuronavigation technology could overcome these drawbacks and maintaining a convenient and immediate translational path to human applications. Neuronavigation is a computer-aided and interactive stereotaxis that localizes the instrument during the session on the neuro-radiologic images acquired before the procedure, and thus allows online feedback for positioning and intra-sessional changes^21–23^. It consists of a position-tracking device to track the positioning of the patient and instruments such as surgical tools and transducers, and an image-processing system to reconstruct and store the images with the information of the instrument location relative to patients^23,24^. During the treatment session, it provides registration between the preliminary images and the physical operating space after calibration based on common features (or fiducials), and guides the operators to position the instruments^23^.

In order to achieve safe and efficient BBB openings, simulation and real-time acoustic monitoring are indispensable. Simulation of acoustic wave propagation^25^ is crucial for planning a patient-specific treatment, as inter- and intra-animal variation of FUS-induced BBB opening has been reported in primates^20^. On the other hand, treatment monitoring for confirming targeting as well as assessing and controlling the treatment outcome dictates treatment precision and time-efficiency. As BBB opening is associated with cavitation (bubble activity such as bubble oscillation and disruption) which can only be monitored acoustically during the treatment, monitoring with passive cavitation detection (PCD)^20,26,27^ and passive acoustic mapping^28–30^ could then ensure the treatment safety and effectiveness while expediting the FUS procedure without the use of the MRI. This can be achieved by real-time passive acoustic mapping guided with neuronavigation in order to confirm the targeting and monitor the treatment in various locations by visualizing the acoustic events in the brain. Therefore, both in silico simulation and real-time acoustic cavitation mapping will be developed and evaluated with *in vivo* BBB opening.

The objective of this study was to develop an efficient transcranial FUS and acoustic mapping system for primates aided by a neuronavigation system. The protocol demonstrated from in silico preplanning and simulation, online treatment and monitoring, to post-treatment assessment for preparation of a clinical trial. The system and protocol were tested in both sedate and awake non-human primates (NHP) with BBB opening to evaluate the performance of simulation, targeting and monitoring accuracy. First, simulation of the transcranial pressure distribution was validated with the *in vivo* BBB opening. Second, the system and protocol for FUS sonication and acoustic mapping was assessed in both a sedate setting, with the animal lying prone on the operating table under anesthesia, and an awake setting, with the animal trained to sit in a customized chair in order to simulate the clinical situation. Lastly, the accuracy of targeting as well as acoustic mapping was evaluated and compared to the frame-based stereotactic method based on contrast-enhanced MRI.

## Results

### Simulation for BBB opening

**Figure 1:**
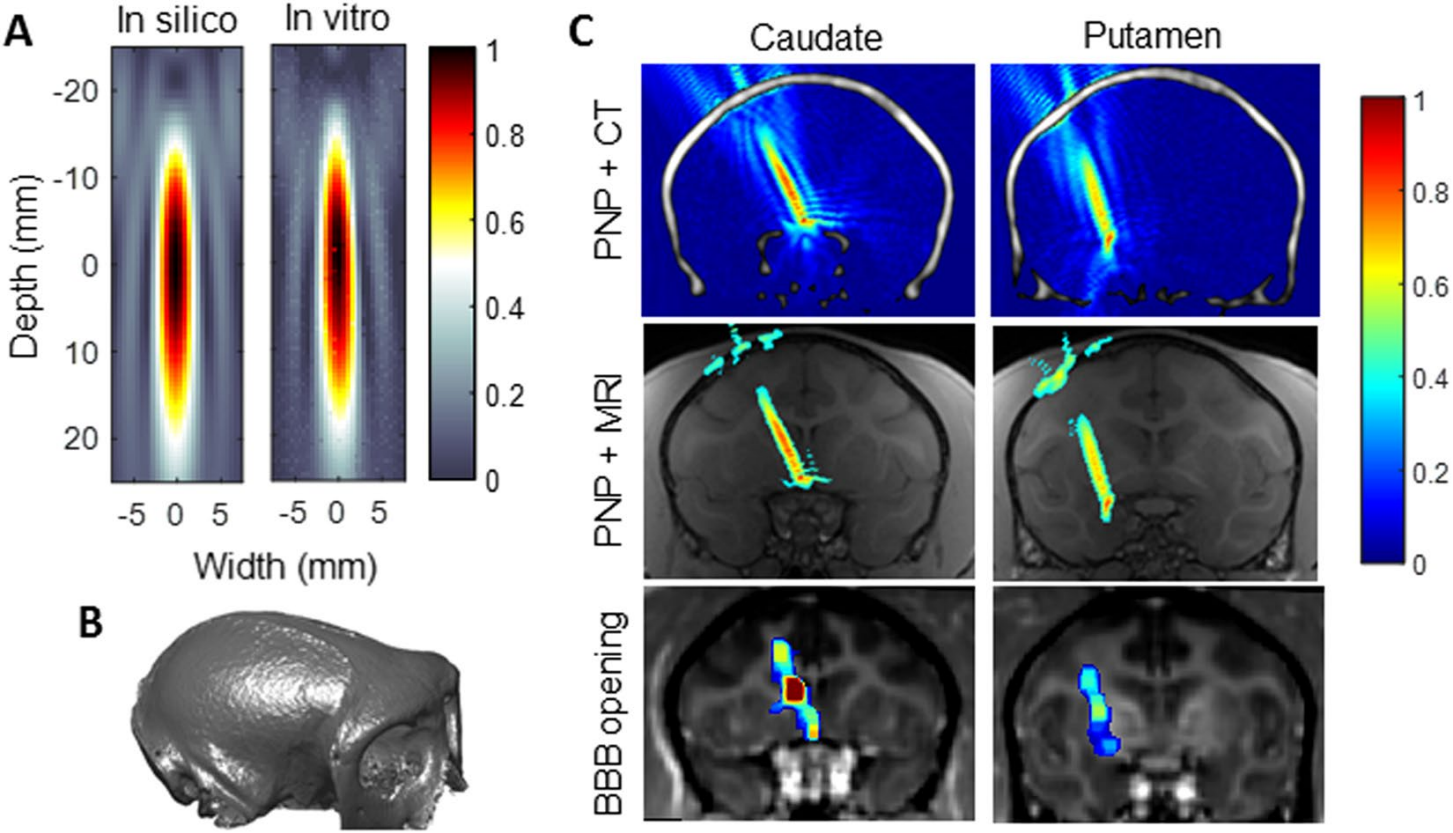
Simulation of the acoustic pressure field to estimate the BBB opening. (**A**) The in silico acoustic profile in the focal region was calibrated to be the same as the profile of the FUS transducer measured in water using a hydrophone. (**B**) The skull of a monkey from CT used to acquire the acoustic properties of the skull including density and the speed of sound. (**C**) The simulated transcranial peak-negative pressure (PNP) field (normalized to the pressure without the skull) corresponded to the BBB opening in the caudate and putamen (arbitrary unit, A.U.).

Since the BBB opening outcome was highly associated with cavitation that is controlled by the acoustic peak-negative pressure (PNP) with circulating microbubbles^20,26^, the transcranial PNP field was simulated and compared with the BBB opening in NHPs *in vivo* (Cohort 1). Before applying the NHP model, the in silico acoustic focus was calibrated with the FUS transducer focus measured in water in terms of the focal length, width, and the sidelobes (Fig. 1A). Then, the NHP model for simulation was constructed based on the CT images in order to acquire the acoustic properties of the skull including the density and the speed of sound (Fig. 1B). It was found that the skull significantly decreased the focal size (the skull lensing effect). The full-width at half maximum (FWHM) focal size based on the acoustic pressure decreased from 4 mm laterally and 35.3 mm axially without the skull to an average of 2.6 mm laterally and 16.7 mm axially through the skull, which was similar to the BBB opening size at 400 kPa. Furthermore, these focal regions were found to be correlated with the BBB opening regions (Fig. 1C) and can be used for personalized preplanning of the FUS treatment.

**Figure 2:**
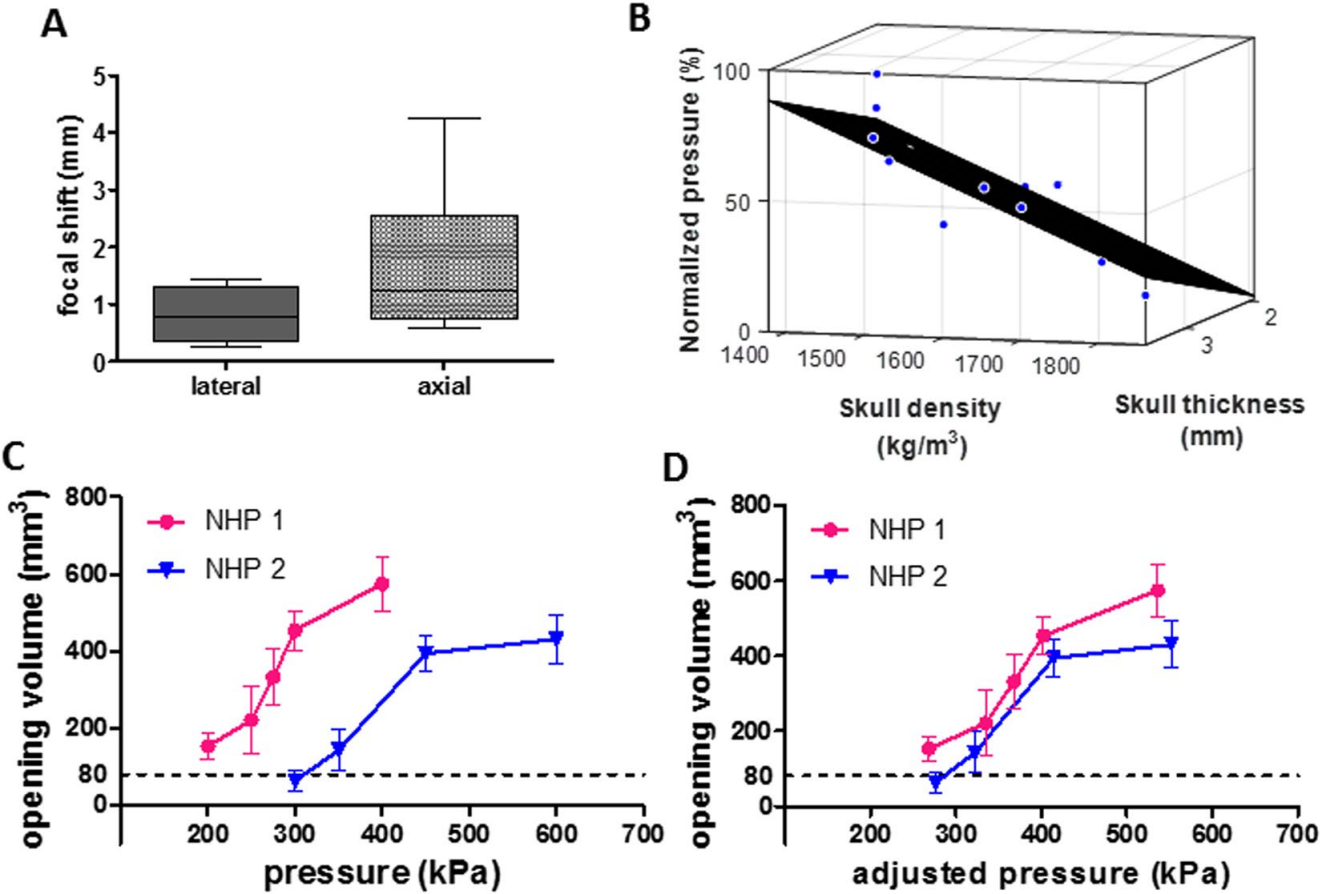
Simulation predicted focal shift and pressure loss due to the skull. (**A**) The focal shift in the lateral and axial direction of the acoustic wave propagation. (**B**) The PNP *in situ* (normalized to that without the skull) was negatively correlated with the thickness and density of the skull in the acoustic beam path. (**C**) Assuming the same pressure loss (50%) resulted in different pressure threshold to induce BBB opening. (**D**) After compensating the pressure loss for each individual based on the simulation (NHP 1: 33%, NHP 2: 54%), the pressure threshold to induce BBB opening became consistent. The error bar in (**C**) and (**D**) represents the standard error of the mean.

Besides the change in the focal size, the skull also affected the location of the focus and the *in situ* pressure. Based on the statistics of the in silico findings in 3 NHPs with 12 targeting, the NHP skull with an average thickness of 2.6 mm, averaged density of 1532 kg/m^3^, and a speed of sound of 2293 m/s resulted in an average focal shift of 2.1 mm (0.8 mm laterally, 1.8 mm axially)(Fig. 2A). The transcranial pressure had an average pressure decrease of 41%, which was varied between animals and targeting and has been found to be highly correlated with the density and thickness of the skull in the beam path (R^2^ = 0.6) (Fig. 2B). This inter-animal variation in the skull attenuation was consistent with the findings on the pressure discrepancy on BBB opening between animals reported previously^20^. After applying various pressures in NHP 1 and 2, a pressure difference was found to induce the same volume of BBB opening (Fig. 2C) assuming a general 50% pressure loss based on *in vitro* calibration. However, based on the in silico measurement the pressure loss due to the skull was 33% in NHP 1 and 54% in NHP 2, which resulted in a similar trend of BBB opening after pressure compensation (Fig. 2D) and the BBB opening threshold was close to what has been reported in small animal models^27,31^. Since the variation in the skull properties could dramatically change the *in situ* pressure and the focal characteristics causing a variation of 300 mm^3^ in BBB opening volume, it is of vital importance to perform pre-treatment simulation for large animals and humans to ensure safety and effectiveness.

### Treatment procedure

**Figure 3:**
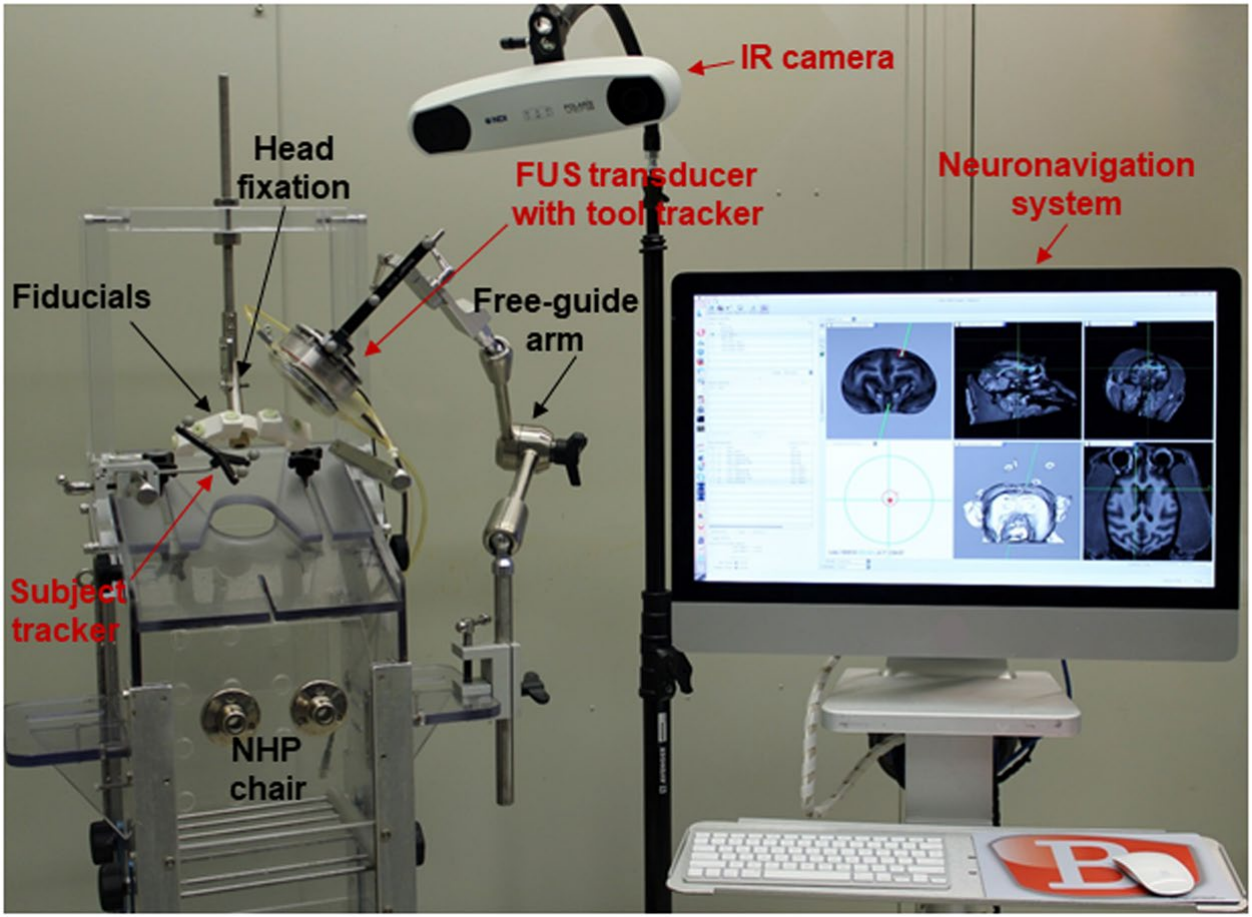
Experimental setup with neuronavigation for the awake animal. Infrared (IR) camera was the position-tracking device connected to the computer to process image registration in real time in the neuronavigation system. The trackers bared three reflective spheres for the IR camera to detect the transducers (with tool trackers) relative to the animal subject in the physical space (with subject tracker). At the beginning of the treatment session, the fiducials were attached to the invariant traits (bite bars or head post) of the animal for registering the animal subject to the neuronavigation system. After the registration, the fiducials were removed and the FUS transducer was aligned to the preplanned targeting and secured with the free-guide arm or stereotactic arm for sonication.

After acquiring the MRI and CT scans of the subject, two experimental groups were designed to compare the targeting accuracy with frame-based stereotaxis^20^ (Cohort 2), to implement computer-aided sonication in the sedate and awake animal setting (Cohort 3) using the setup shown in Fig. 3. The experimental cohort 2 (7 sonications in 2 NHPs) was planned with the stereotactic calculation, and the location of the acoustic focus was visualized and recorded in the neuronavigation system during the FUS session. The experimental cohort 3 (15 sonications in 3 NHPs) was planned on the neuronavigation software before sonication, and the targeting was implemented through the guidance of the neuronavigation system during the sonication session. This cohort demonstrated the feasibility of computer-aided FUS sonication. The computer-aided acoustic mapping was performed in all cohorts. Both the targeting accuracy and the acoustic mapping corresponded to BBB opening were quantified in all cohorts.

In the preplanning process on the neuronavigation system, the 3D skull, subcortical structure, the scalp was segmented and visualized together with the MRI slices in order to assist the selection of the focal region and the trajectory by covering the region of interest while avoiding pre-existing lesions, large vessels, ventricles inside the brain, as well as physical hindrance such as implants, craniotomy due to previous surgeries, or thick epicranial muscle in the beam path outside the brain. The selected targeting could then be exported for simulation to estimate the pressure distribution *in situ*.

**Figure 4:**
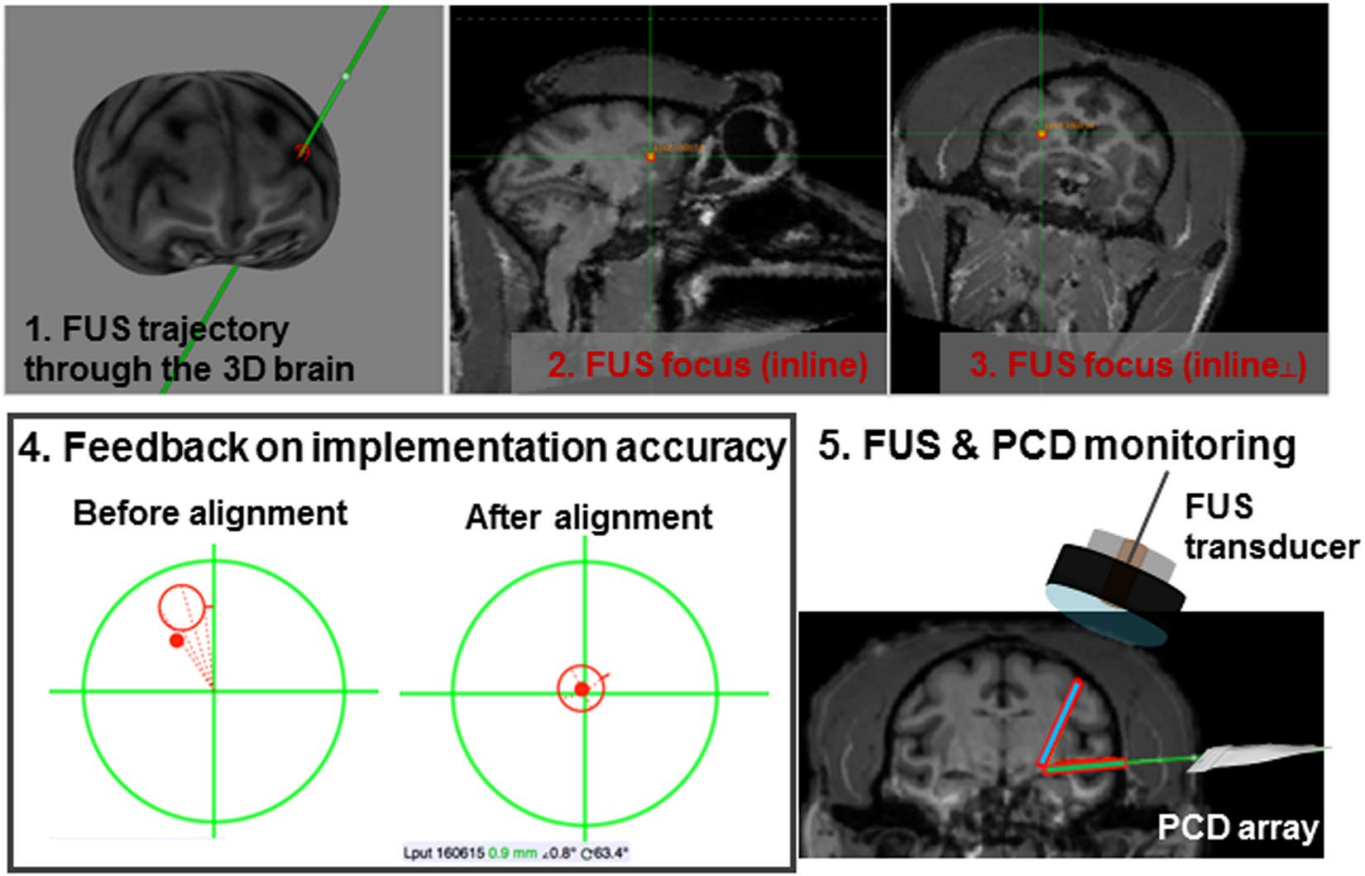
Screenshots of the neuronavigation-guided FUS session. Online session of the FUS procedure showing the FUS trajectory in the reconstructed 3D brain (1) targeted the putamen in two orthogonal MRI slices in line with the FUS transducer, where the vertical arrows represent the FUS trajectory pointing at the focus (2–3). (4) The implementation accuracy of the FUS transducer to the preplanning was displayed as a feedback for the distance (visualized as the distance between the red dot and the center of the larger circle) and the angle deviation (visualized as the distance between the red dot and the center of the smaller circle) during the guiding process. (5) The monitoring transducer for acoustic mapping was aligned to the FUS focus before sonication with neuronavigation guidance.

During the treatment session, both the FUS transducer and the monitoring transducer were guided and aligned with the neuronavigation system, with the screenshots shown in Fig. 4. The subject was first registered to the preliminary anatomical images based on the doughnut-shaped fiducials in order to create a linkage between the physical space and the virtual image space shown on the computer screen, and the error for each fiducial was kept below 1.5 mm after registration. Followed by the installation of the transducer with the free-guide mechanical arm, the trajectory of the FUS beam and the focus were visualized in the virtual image space in real time with feedback on the targeting implementation accuracy (Step 4 of Fig. 4). Specifically, the distance and angular deviation was both listed on the panel interface and reflected graphically by the distance of the red dot to the center of the green circle and to the red circle, respectively, which gave two concentric circles for a perfect alignment. This alignment accuracy in session, i.e., the distance and angle deviation of the FUS beam to the preplanned targeting were respectively kept below 1 mm and 5°. After the FUS transducer was set to the preplanned targeting orientation, the monitoring transducer was then placed against the temporal bone toward the FUS focus with the imaging plane covering the focus through the neuronavigation guidance (Step 5 of Fig. 4). At the beginning of the sonication, the sonicated region was confirmed with the real-time cavitation mapping, and then the BBB opening was monitored throughout the entire sonication. The positions of the two transducers were recorded for offline processing of the targeting and monitoring accuracy compared with the BBB opening results. The entire procedure from registration of the subject (5 to 10 min), neuronavigation of the FUS beam and the monitoring probe (10 to 15 min), to the sonication (2 min) lasted around 30 min for awake animal experiments, and 30–60 min for sedate animal experiments that required stereotaxis for head fixation.

### Accuracy of neuronavigation-guided sonication and monitoring

**Figure 5:**
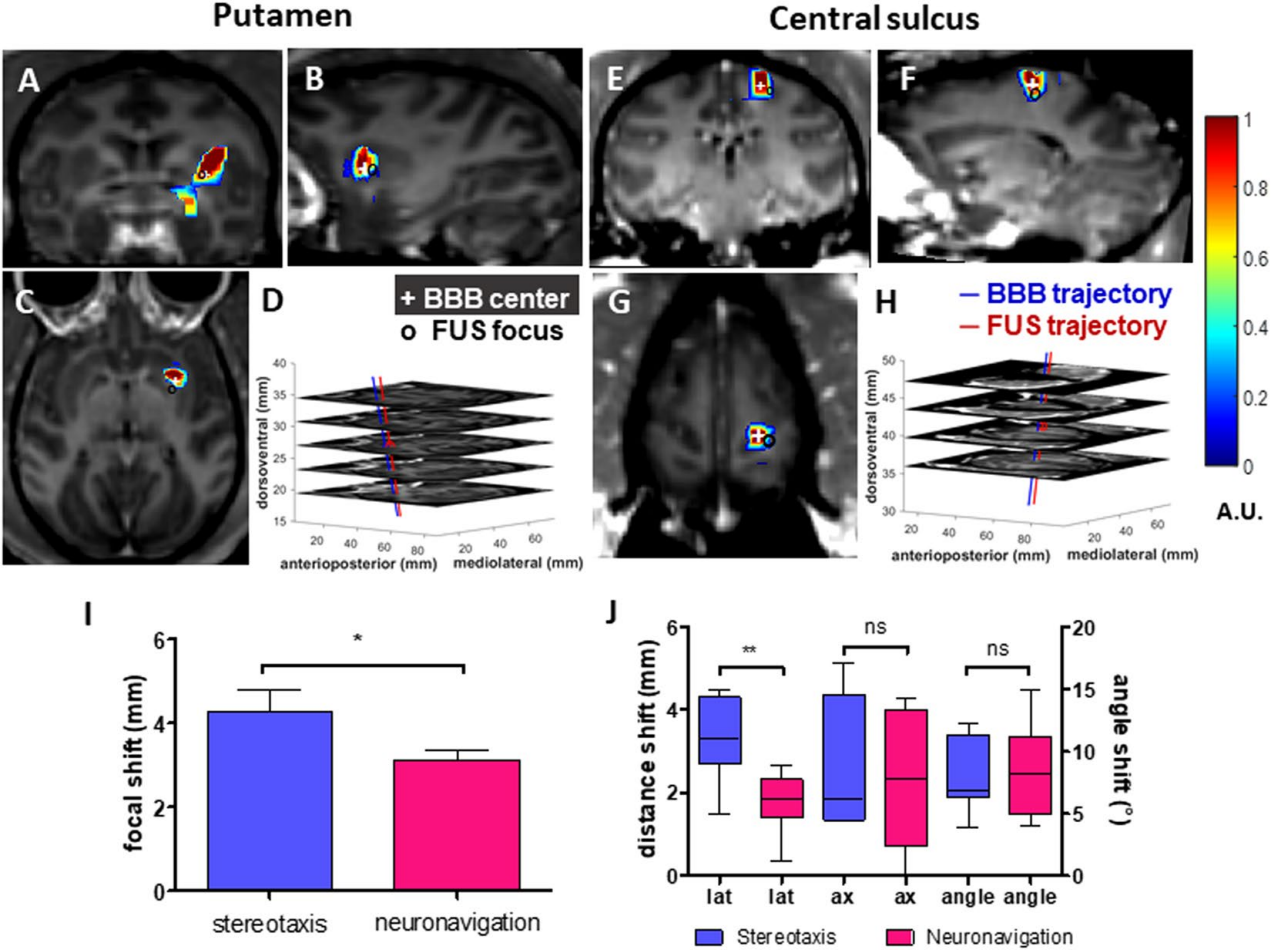
Neuronavigation-guided FUS for BBB opening and targeting accuracy in monkeys. (**A**–**D**) BBB opening in the basal ganglia in the sedate animal. (**E**–**H**) BBB opening in the primary motor cortex in the awake animal. Coronal: (**A**) and (**E**); sagittal: (**B**) and (**F**); horizontal: (**C**) and (**G**). (**D**) and (**H**): the stacked horizontal slices with the BBB opening trajectory (red line), the planned trajectory (blue line), and the centroid of BBB opening and the FUS focus shown in the neuronavigation system were denoted in ‘ + ’ and ‘o’, respectively. (**I**) Accuracy measurement showed that the total focal shift with the neuronavigation was smaller than with the stereotaxis (two-tailed Student’s t-test). (**J**) After breaking into the lateral (lat), the axial (ax) direction and the angle, the neuronavigation showed a significant improvement on the lateral direction (one-way ANOVA). The error bar represents the standard error of the mean. *p < 0.05. **p < 0.01.

Both the basal ganglia and the cerebral cortex were targeted with successful BBB opening through neuronavigation guidance. Figure 5 shows the representative BBB opening results in the sedate (Fig. 5A–D) and awake (Fig. 5E–H) animal setup, and the quantitative targeting accuracy (Fig. 5I,J) was calculated as the deviation between the recorded focus and FUS trajectory in the navigation system and the BBB opening trajectory based on the center of mass and line fitting in the MRI results. The average accuracy of neuronavigation was equal to 3.1 mm, which was better than the frame-based stereotaxis (4.3 mm) in the NHP model (Fig. 5I), and close to the predicted transcranial focal shift in ultrasound wave propagation (2.1 mm) (Fig. 2A). The lateral shift of the FUS beam was found to be significantly decreased, from 3.2 mm to 1.8 mm, with the use of neuronavigation while the axial shift and angular shift remained within the same range (2.6–3.2 mm and 8°−9°). The targeting accuracy in the awake animal setting (3.0 mm) was comparable to the sedate animal setting (3.2 mm). Overall, the accuracy of the computer-aided FUS was consistent to what has been reported in the neuronavigation-guideded surgery in humans (1.6–3.0 mm)^21^.

**Figure 6:**
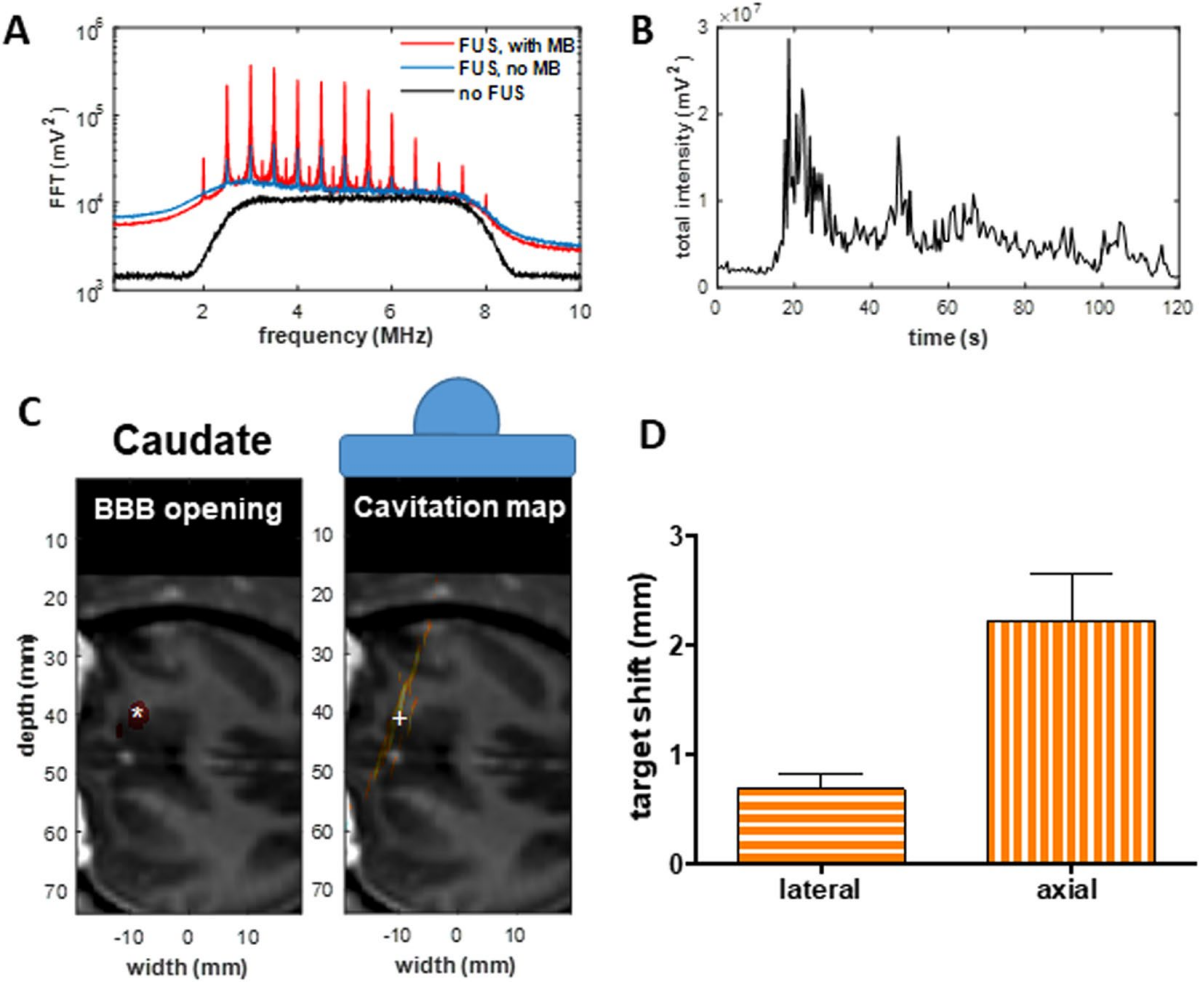
Neuronavigation-guided cavitation detection and mapping during sonication for BBB opening at 450 kPa. (**A**) The average frequency spectra in the channel data of the PCD array. (**B**) The total intensity of the channel data during the 2-min sonication showed significant cavitation response after microbubbles perfused the brain 20 s after microbubble injection. The reconstructed cavitation maps showed the exact location of the BBB opening in the caudate (**C**), where ‘*’ represents the centroid of BBB opening and ‘ + ’ represents the centroid of the cavitation event. (**D**) Distance between the cavitation events in the cavitation maps and the centroid of BBB opening showed an accurate monitoring at the sonicated region. The error bar represents the standard error of the mean.

Real-time cavitation mapping was performed with neuronavigation guidance during the entire sonication, and monitoring results with the corresponding BBB opening were shown in Fig. 6. The average frequency spectra in a single pulse of the acquired channel data showed a dramatic increase of the cavitation signal (harmonics and ultraharmonics) after injecting microbubbles (Fig. 6A), and the total acoustic signal intensity (sum of the squared cavitation signal amplitude in each pulse) indicated the persistence of microbubbles over the entire 2-min sonication duration in the brain (Fig. 6B). In the cavitation maps, the location of the cavitation event during sonication overlapped with the BBB opening region as shown in the caudate (Fig. 6C). The overall monitoring accuracy (Fig. 6D) showed an average distance of 2.4 mm between the centroid of the BBB opening volume and the cavitation map (0.7 mm laterally and 2.2 mm axially), with no significant difference between animals or targeted regions.

In all studies presented, no acute damage such as hemorrhage (SWI) or edema (T2-weighted imaging) was detected upon radiologic examination 2 h after sonication.

## Discussion

In this study, a subject-specific and time-efficient transcranial FUS and monitoring system with high precision was developed and demonstrated from in silico preplanning to BBB opening with real-time targeting guidance and acoustic mapping in both sedate and awake non-human primates. For the first time, a noninvasive FUS treatment was achieved in a brief procedure time (30 min) without an online MRI constraint. This duration was deemed acceptable for patients since it was similar to most clinical imaging procedures. The targeting flexibility of our system allowed BBB opening in both the cerebral cortex and deeply-seated subcortical structures with the use of a free-guide arm and the inflatable bladder system of the FUS transducer to couple with the scalp. Its application will benefit a broad patient population especially with diseases requiring repeated procedure and customized treatment, which is unprecedented of current systems. It will also facilitate the FUS neuromodulation research in primates. Furthermore, real-time monitoring for the energy distribution of the acoustic cavitation in combination with the neuronavigation technology holds great potential to assess and control the treatment in the targeted regions.

This neuronavigation-guided ultrasound system tested in non-human primates maintains translational capability to a clinical setting. The demonstrated protocol encompasses in silico preplanning, real-time targeting procedure and monitoring, to post-treatment assessment. First, in the initial preplanning after acquiring the MRI with fiducials and CT, the region of interest and trajectory for the acoustic beam could be selected in the neuronavigation panel in the preplanning phase assisted by the 3D reconstructed brain structure and scalp. After the focus and trajectory are chosen, the transcranial pressure field could be simulated to estimate the focal size, focal shift, and the *in situ* pressure through the skull. Second, once optimal targeting has been placed, the neuronavigation system can be adjusted for sonication and cavitation mapping in real time. Finally, the treatment outcome is evaluated. For BBB opening and drug delivery, the animal is currently evaluated with contrast-enhanced MRI using gadodiamide (10 min.). This may no longer be required with the use of acoustic monitoring^20^.

This computer-aided system holds several advantages compared with other clinical systems such as MRgFUS^2,32,33^ and the implantable ultrasound device^14^, and were summarized in Table 1 ^24,34,35^. In contrast to MRgFUS whose procedure may take 3 h and the targeting is limited due to the fixed transducer orientation^15,17,33^, the fast procedure and targeting flexibility of our system could facilitate translation to the clinic, and benefit a significantly larger patient population while maintaining the required treatment precision. Our technique also enables treatment modification if different targeted regions or larger BBB opening region (e.g. tumor progression) are desired, a tremendous advantage over the implantable and unfocused ultrasound device. Our system is thus suitable for both preclinical and clinical applications based on the non-thermal (mechanical) effects that require iterative treatment such as BBB opening, drug delivery, and neuromodulation. On the other hand, the safety is considered to be mainly dependent upon the acoustic parameters and dosage of microbubbles used^36,37^, and less affected by the system used.

**Table 1:** Comparison for current ultrasound systems used in the brain.

Ultrasound system	MR guided	Neuronavigation guided	Implantable device
Invasiveness	Noninvasive	Noninvasive	Invasive
Targeting method	MR thermometry with mild temperature increase in the tissue	Pre-registration between pre-MRI and physical space based on common features	Not required
Targeting precision	1 mm (ablation)	2 mm	Not applicable
Room time	3 h	0.5 h	10 min
Transducer requirement	MR compatible	No constraint	Tissue compatible
Space requirement	Dedicated MRI suite	No constraint	No constraint
Cavitation monitoring	Applicable	Applicable	Not applicable
Applications	Surgical ablation, drug delivery, stimulation	Drug delivery, stimulation	Drug delivery, stimulation

This technology also advances upon the previously developed frame-based stereotactic method with single-element cavitation monitoring in several folds. First, the online visualization and guidance allowed an interactive adjustment for accurate targeting due to the online feedback. The frame-based stereotactic method lacks feedback on positioning during sonication, and the positioning was subject to the anatomy of ear canals with the use of ear bars. Therefore, any slight movement of the animal or transducer in the stereotactic frame introduces targeting variation in this study. Second, its flexibility in preplanning and implementation through the free-guide mechanical arm grants higher degrees of freedom for transducer placement, and enables the transducer orientation to align with the brain structure^38^, which is not provided through the stereotaxic arm. Third, cavitation mapping combined with neuronavigation visualizes the spatial distribution of cavitation events associated with the treatment outcome. This is advantageous over the previous system^24^ as it will allow for confirmation of the targeting, monitoring and assessment of the treatment in real time. However, this monitoring technique is independent of the FUS targeting so it did not improve the targeting accuracy. Finally, this system greatly facilitates both preclinical studies in BBB opening with awake sonication in primates. It will benefit the study for neuromodulation and ease the clinical translation and widespread use of BBB opening.

The targeting accuracy was determined by errors in the neuronavigation system^39^, skull distortion of the acoustic wave propagation, and the BBB opening heterogeneity^20^. On the other hand, the monitoring accuracy was affected by both the neuronavigation system and cavitation mapping acquired through the skull and its resolution. With the neuronavigation system, the targeting accuracy in the lateral direction was significantly improved compared to the frame-based stereotaxis. This improvement could be due to the variability involved in the animal placement within the stereotaxic frame with the ear bars. However, the axial shift and angle shift remained identical and may be due to the skull based on the simulation results or the characteristics of BBB opening as the gray matter had higher probability of BBB opening than the white matter^20^. As shown in Fig. 2A and 8B, an axial focal shift of 1.8 mm was estimated in silico, which was similar to the axial shift of 2.4 mm with neuronavigation. For the acoustic mapping, on the other hand, although the skull could also affect the accuracy in terms of location and focal quality of the cavitation events due to phase aberration^40^, the effect was minimal through the temporal bone in this study. A more accurate monitoring assessment in the lateral direction in comparison to the axial direction could be due to a better mapping resolution in the lateral direction.

There are limitations and ways for improvement of the proposed system. Sources of error in targeting are caused by registration errors with the neuronavigation system, the focal shift due to the skull, and the error in post-processing of the MRI, etc. To minimize the registration error, the location of the fiducials during treatment should be invariant to the preliminary MRI with fiducials. In addition, since the neuronavigation system localized the tools relative to the subject tracker, maintaining a distance between the subject tracker and target as small as possible (<10 cm) could improve the accuracy. To reduce the acoustic focal shift due to the skull, simulation of the acoustic pressure field and the use of phased array focused ultrasound transducer with phase aberration correction could potentially compensate this type of error. Lastly, in order to evaluate or control the treatment safety and efficacy, a robust acoustic mapping technique is required to minimize the need for an MRI as a tool for treatment assessment. In this study, the monitoring transducer was placed proximal to the thinnest temporal bone to minimize the skull effects, which also took one extra procedure aligning it with the FUS transducer. With the monitoring transducer concentric to the FUS transducer it will save effort in the procedure, but the monitoring performance may be more affected by the skull. In the future, the performance of using a monitoring transducer concentric to the FUS transducer will be assessed and compared for optimization purposes.

## Material and Methods

### Computer-aided ultrasound system

An arm-free, real-time neuronavigation system (Brainsight Vet System, Rogue Research Inc., Canada) designed for primates (both monkeys and humans) was customized to be used in conjunction with an ultrasound system. This neuronavigation system was based on paired-point registration with an optical tracking device and reflective spheres (Northern Digital Inc., CA, USA), and the fiducial bite bar system (bite bars bearing six fiducials; Rogue Research Inc., Canada) were constructed for each individual with their unique tooth imprints in the sedate animal experiments. For the awake animal experiments, in-house fiducial bearing pieces were designed attachable to the head post implantation of the animal. Two unique tool trackers with three reflective spheres were mounted separately on each transducer (Fig. 3) and calibrated for the neuronavigation system in order to recognize them in the physical space. The ultrasound system consisted of a FUS treatment unit controlled by a customized program in Matlab with a single-element, 0.5-MHz FUS transducer (diameter: 64 mm, focal depth: 62.6 mm; H-107, Sonic Concepts, WA, USA) triggered by a function generator (model 33220 A, Agilent Technologies, CA, USA) after 50-dB amplification (A075, ENI, NY, USA), and an acoustic monitoring unit with a programmable acoustic signal acquisition system (Vantage 256, Verasonics, WA, USA) and an array of acoustic detectors (Philips ATL L7–4 linear array, bandwidth = 2 to 8 MHz, 38 mm wide with 128 elements) synchronized with the FUS system for real-time passive acoustic mapping and storage of the entire acoustic signals. Both the FUS and acoustic mapping were guided with the neuronavigation system during the entire FUS procedure.

### Experimental design

In accordance with the National Institutes of Health Guidelines for animal research, all procedures and experiments were reviewed and approved by the Institutional Animal Care and Use Committee at Columbia University and the New York State Psychiatric Institute. Four male adult macaques (3 Macaca mulatta and 1 Macaca fascicularis, weight: 6–11 kg, age: 8–20 yo) sonicated at 0.3, 0.45, and 0.6 MPa (excitation frequency = 0.5 MHz, pulse length = 10 ms, pulse repetition frequency = 2 Hz, duration = 2 min) with in-house microbubbles injected intravenously (lipid-shelled, 4–5 µm in diameter, 2.5 × 10^8^ bubbles/kg)^26^, and targeted structures included the basal ganglia (caudate and putamen) associated with neurodegenerative diseases such as Parkinson’s and Huntington’s disease as well as the primary motor cortex in the central sulcus and precentral gyrus. Three experimental groups (cohorts) were designed. The sonications were performed in different locations independently in order to assess the variability of targeting accuracy in the primate brain as a part of the system performance. First, stereotaxic sonication (NHP 1, 2, 3) with stereotaxic planning^18,41,42^ was for BBB opening in validation of the acoustic pressure field simulation (N = 36). Second, computer-aided sonication (NHP 2, 3, 4) were performed in cohort 2 (N = 7) and 3 (N = 15) to assess the targeting accuracy. Specifically, neuronavigation in conjunction with stereotaxic sonication (NHP 2, 3) in cohort 2 to investigate the targeting and monitoring accuracy by locating the acoustic focus with neuronavigation with stereotaxic preplanning. In cohort 3, computer-aided sonication (NHP 2, 3, 4) with preplanning on the neuronavigation system was implemented to investigate the targeting and monitoring accuracy through neuronavigation guidance in both the sedate and awake animal settings. The computer-aided sonication was translated to an awake animal setting in order to mimic the clinical settings, where the animal (NHP 4) was trained to sit in a customized chair with its head stabilized by an attachment to the chair. A total of 58 sonications (independent experiments) were performed in this study.

### Preliminary image acquisition

Both computerized tomography (CT) and magnetic resonance imaging (MRI) were acquired before the FUS treatment for personalized preplanning and neuronavigation guidance. CT (helical scan, resolution = 0.2 × 0.2 × 0.6 mm^3^; Siemens) was used to extract the skull properties such as density and thickness in order to estimate the acoustic energy loss in simulation, and T1-weighted MRI (3D turbo field echo sequence, TR/TE = 11.1/5.1 ms, FA = 8°, resolution = 0.7 × 0.7 × 0.7 mm^3^; Philips 3 Tesla scanner) for the anatomical scan of the brain surrounded by six contrast-enhanced fiducials used for registration of the neuronavigation system.

### Acoustic wave simulation

In order to estimate the focal shift and the acoustic pressure decrease due to the skull, the 3D k-space pseudospectral method (k-Wave)^25^ was used to simulate the acoustic wave propagation and the peak-negative pressure field in solving the coupled first-order system of wave equations. The ring-shaped focused transducer constituted the acoustic source with the focal size calibrated based on the FUS transducer calibration in water at room temperature, with the same excitation frequency and pulse length used for the *in vivo* experiments. The acoustic properties of the skull including the speed of sound and density were converted from the Hounsfield units in CT^43^, with an attenuation of 20 dB/cm and the power law absorption exponent of 1.1 based on previous measurements^44,45^. The medium properties surrounding the skull were the same as water at the body temperature (37 °C, speed of sound = 1524 m/s, density = 1000 kg/m^3^, attenuation = 3.5 × 10^−4^ dB/cm).

### Reconstruction of acoustic maps

The channel data of all the cavitation signals were acquired and processed during the sonication, and a total of 240 pulses were saved in the computer at the end of the sonication for offline analysis as well. Time-exposure acoustics^28,29^ in combination with parallel beamforming, performed by multiplying a reconstruction sparse matrix to the radiofrequency channel data on a graphic processing unit^46^ (GPU; Tesla K40, NVIDIA), were developed for the reconstruction of passive acoustic maps in real time during the sonication (frame rate = pulse repetition rate = 2 Hz). In a single pulse, one cavitation map was generated by summing up the 30 passive image frames (root-mean-square amplitude of the radiofrequency signals in frames) been reconstructed as an integration over an exposure time of 1.44 µs to enhance the cavitation signal while eliminating any electronic noise. Each cavitation map was constructed within 0.5 s of pulse repetition time in order to be monitored in real time. On the other hand, an exposure time of 62.5 µs was used for off-line processing in order to acquire the optimal acoustic mapping quality for post-comparison to the BBB opening.

### Experimental procedure

The experimental setup is shown in Fig. 3. For animal under anesthesia, the animal was sedated with ketamine (10 mg/kg in conjunction with 0.02–0.04 mg/kg of atropine through intramuscular injection) for placement of an endotracheal tube and an intravenous catheter in the saphenous vein, and then transported to a dedicated suite for the anesthesia procedure (1–2% isoflurane-oxygen mixture) with vital signs monitored during the entire experiment^18,19^. While for the awake animal experiments, the animal was lightly sedated with ketamine (5 mg/kg) for placement of an intravenous catheter in the saphenous vein prepared for injection of microbubbles, and then was placed into the chair with head fixed while awake^47^. During the FUS session, the animal subject in the physical space (represented by the subject tracker) was first registered to the virtual image space on the neuronavigation system. Specifically, the pointer tool recognized by the system was used to select the fiducials one-by-one, which were bared on the bite bar for sedate animals secured by the upper jaw of the animal after been fixed on the stereotaxic frame or on the head post for the awake animal. Once registered, the orientation of the tools (represented by the tool trackers) relative to the brain including the FUS transducer and the imaging probe could be displayed on the real-time reconstructed 2D and 3D images on the neuronavigation monitor. In cohort 2 (neuronavigation in conjunction with stereotaxic sonication), the stereotaxic targeting^18,20^ was visualized and recorded on the neuronavigation system. In cohort 3 (computer-aided sonication), the mechanical arm was utilized to align the FUS transducer to the preplanned targeting in terms of the focus and orientation in both the sedate and awake animal setup. For the awake animal setting, the animal was secured in the customized chair for computer-aided sonication. In cohort 2 to 3, the monitoring transducer for acoustic mapping was aligned to the FUS focus against the temporal bone, the thinnest part of the skull with less acoustic signal attenuation. In order to show the initial monitoring feasibility, the cavitation mapping data were acquired at different locations in one sonication to verify the accuracy of localization. Specifically, the monitoring transducer was placed at 2–3 locations within 1 cm, and each location was recorded in the neuronavigation system for offline assessment. Since the FUS transducer targeted the same spot in one single sonication, the procedure did not affect the BBB opening.

At the beginning of the sonication, the microbubbles were injected in a bolus intravenously followed by saline flush within 30 s, and the acoustic maps were displayed in real time during the entire sonication. In order to confirm the BBB opening and safety, MRI (3 T) was performed 1 h after sonication and compared with the baseline scans before sonication^20^. T_1_-weighted images before and after gadolinium injection (Gd-DTPA-BMA, Omniscan®, GE Healthcare, NJ, USA; 0.2 mL/kg) for confirming BBB opening (3D spoiled gradient echo sequence, TR/TE = 8.5/4.8 ms, FA = 8°, resolution = 1 × 1 × 1 mm), T_2_-weighted images for assessing potential edema (TR/TE = 3000/80 ms, flip angle or FA = 90°, resolution = 0.4 × 0.4 × 2 mm), and susceptibility-weighted images for assessing potential hemorrhage (TR/TE = 19/27 ms, FA = 15°, resolution = 0.4 × 0.4 × 1 mm).

### Accuracy analysis of targeting and acoustic mapping

In analyzing the targeting accuracy for the BBB opening, the contrast enhancement from the sonicated region was first identified by taking a division of the post- to the pre-contrast T_1_-weighted images and filtering the vessel signal with the control scan (pre- and post-contrast T_1_-weighted images without sonication) as described previously^20^. The center and the trajectory of the BBB opening was defined as the center of mass in 3D and the linear fit of the center of mass in each 2D slices in the volume of interest (10 × 10 × 32.5 mm^3^), which was compared with the location and trajectory of the ultrasound focus recorded by the neuronavigation system in order to assess the target shift.

For analyzing the monitoring accuracy, the enhancement image with BBB opening that corresponded to the imaging plane of the monitoring probe was interpolated based on the pixel position in the 3D brain images, which was calculated based on the transformation matrix provided by the neuronavigation system. The centroid of the cavitation maps was then calculated and compared with the centroid of the BBB opening in the enhancement image in order to estimate the distance shift.

## References

[1] World-Health-Organization (ed.) Neurological disorders: public health challenges, Edn. 1st. (WHO Press, 2006).

[2] Jolesz FA (2009). MRI-guided focused ultrasound surgery. Annu Rev Med.

[3] Leinenga G, Langton C, Nisbet R, Gotz J (2016). Ultrasound treatment of neurological diseases - current and emerging applications. Nat Rev Neurol.

[4] Timbie, K.F., Mead, B.P. & Price, R.J. Drug and gene delivery across the blood-brain barrier with focused ultrasound. *J Control Release* (2015). 10.1016/j.jconrel.2015.08.059 PMC4656107 26362698

[5] Leinenga G, Gotz J (2015). Scanning ultrasound removes amyloid-beta and restores memory in an Alzheimer’s disease mouse model. Sci Transl Med.

[6] Burgess A (2014). Alzheimer disease in a mouse model: MR imaging-guided focused ultrasound targeted to the hippocampus opens the blood-brain barrier and improves pathologic abnormalities and behavior. Radiology.

[7] Liu HL (2010). Blood-brain barrier disruption with focused ultrasound enhances delivery of chemotherapeutic drugs for glioblastoma treatment. Radiology.

[8] Kinoshita M, McDannold N, Jolesz FA, Hynynen K (2006). Noninvasive localized delivery of Herceptin to the mouse brain by MRI-guided focused ultrasound-induced blood-brain barrier disruption. Proc Natl Acad Sci USA.

[9] Legon W (2014). Transcranial focused ultrasound modulates the activity of primary somatosensory cortex in humans. Nat Neurosci.

[10] Tufail Y, Yoshihiro A, Pati S, Li MM, Tyler WJ (2011). Ultrasonic neuromodulation by brain stimulation with transcranial ultrasound. Nat Protoc.

[11] Elias WJ (2013). A pilot study of focused ultrasound thalamotomy for essential tremor. N Engl J Med.

[12] FUS-Foundation (ed.) Focused Ultrasound State of the Field, Vol. 1, Edn. 1. (Focused Ultrasound Foundation, 2016).

[13] Schlesinger I (2015). MRI Guided Focused Ultrasound Thalamotomy for Moderate-to-Severe Tremor in Parkinson’s Disease. Parkinsons Dis.

[14] Carpentier A (2016). Clinical trial of blood-brain barrier disruption by pulsed ultrasound. Sci Transl Med.

[15] FUS-Foundation (ed.) Focused ultrasound for Alzheimer’s disease workshop summary (2015).

[16] McDannold, N., Clement, G.T., Black, P., Jolesz, F. & Hynynen, K. Transcranial magnetic resonance imaging- guided focused ultrasound surgery of brain tumors: initial findings in 3 patients. *Neurosurgery* **66**, 323–332 discussion 332 (2010). 10.1227/01.NEU.0000360379.95800.2F PMC2939497 20087132

[17] FUS-Foundation (ed.) Focused ultrasound for glioblastoma workshop summary. (2015).

[18] Marquet F (2014). Real-time transcranial monitoring of safe blood-brain barrier opening in non-human primates. PLoS ONE.

[19] Wu SY (2014). Transcranial cavitation detection in primates during blood-brain barrier opening–a performance assessment study. IEEE Trans Ultrason Ferroelectr Freq Control.

[20] Wu, S.-Y. *et al*. Characterizing Focused-Ultrasound Mediated Drug Delivery to the Heterogeneous Primate Brain *In Vivo* with Acoustic Monitoring. *Sci Rep* **6** (2016). 10.1038/srep37094 PMC5112571 27853267

[21] Spetzger U, Laborde G, Gilsbach JM (1995). Frameless neuronavigation in modern neurosurgery. Minim Invasive Neurosurg.

[22] Ganslandt O, Behari S, Gralla J, Fahlbusch R, Nimsky C (2002). Neuronavigation: concept, techniques and applications. Neurol India.

[23] Grunert, P., Darabi, K., Espinosa, J. & Filippi, R. Computer-aided navigation in neurosurgery. *Neurosurg Rev* **26**, 73–99 discussion 100-101 (2003). 10.1007/s10143-003-0262-0 12962294

[24] Wei KC (2013). Neuronavigation-guided focused ultrasound-induced blood-brain barrier opening: a preliminary study in swine. AJNR Am J Neuroradiol.

[25] Treeby BE, Jaros J, Rendell AP, Cox BT (2012). Modeling nonlinear ultrasound propagation in heterogeneous media with power law absorption using a k-space pseudospectral method. J Acoust Soc Am.

[26] Wu SY, Chen CC, Tung YS, Olumolade OO, Konofagou EE (2015). Effects of the microbubble shell physicochemical properties on ultrasound-mediated drug delivery to the brain. J Control Release.

[27] O’Reilly MA, Hynynen K (2012). Blood-Brain Barrier: Real-time Feedback-controlled Focused Ultrasound Disruption by Using an Acoustic Emissions-based Controller. Radiology.

[28] Norton SJ, Won IJ (2000). Time exposure acoustics. Ieee T Geosci Remote.

[29] Gyongy M, Coussios CC (2010). Passive spatial mapping of inertial cavitation during HIFU exposure. IEEE Trans Biomed Eng.

[30] Arvanitis CD, Livingstone MS, McDannold N (2013). Combined ultrasound and MR imaging to guide focused ultrasound therapies in the brain. Physics in Medicine and Biology.

[31] Tung YS, Vlachos F, Feshitan JA, Borden MA, Konofagou EE (2011). The mechanism of interaction between focused ultrasound and microbubbles in blood-brain barrier opening in mice. J Acoust Soc Am.

[32] McDannold N, Arvanitis CD, Vykhodtseva N, Livingstone MS (2012). Temporary disruption of the blood-brain barrier by use of ultrasound and microbubbles: safety and efficacy evaluation in rhesus macaques. Cancer research.

[33] McDannold N (2016). Preclinical evaluation of a low-frequency transcranial MRI-guided focused ultrasound system in a primate model. Phys Med Biol.

[34] Chauvet D (2013). Targeting accuracy of transcranial magnetic resonance-guided high-intensity focused ultrasound brain therapy: a fresh cadaver model. J Neurosurg.

[35] Moser D, Zadicario E, Schiff G, Jeanmonod D (2013). MR-guided focused ultrasound technique in functional neurosurgery: targeting accuracy. J Ther Ultrasound.

[36] McMahon D, Hynynen K (2017). Acute Inflammatory Response Following Increased Blood-Brain Barrier Permeability Induced by Focused Ultrasound is Dependent on Microbubble Dose. Theranostics.

[37] Shin J (2018). Focused ultrasound-mediated noninvasive blood-brain barrier modulation: preclinical examination of efficacy and safety in various sonication parameters. Neurosurg Focus.

[38] Deffieux T, Konofagou EE (2010). Numerical study of a simple transcranial focused ultrasound system applied to blood-brain barrier opening. IEEE Trans Ultrason Ferroelectr Freq Control.

[39] Wang MN, Song ZJ (2011). Classification and analysis of the errors in neuronavigation. Neurosurgery.

[40] Jones RM, O’Reilly MA, Hynynen K (2015). Experimental demonstration of passive acoustic imaging in the human skull cavity using CT-based aberration corrections. Med Phys.

[41] Marquet, F., Tung, Y.-S., Teichert, T., Ferrera, V. P. & Konofagou, E. E. Non-invasive, transient and selective blood-brain barrier opening in non-human primates *in vivo*. *PLoS One*, **6**(7), e.22598 (2011). 10.1371/journal.pone.0022598 PMC3142168 21799913

[42] Tung, Y.-S., Marquet, F., Teichert, T., Ferrera, V. P. & Konofagou, E. E. Feasibility of cavitation-guided blood-brain barrier opening using FUS and microbubbles in non-human primates. *Applied Physics Letters*, **98**(16), e.163704 (2011). 10.1063/1.3580763 PMC3094460 21580802

[43] Schneider U, Pedroni E, Lomax A (1996). The calibration of CT Hounsfield units for radiotherapy treatment planning. Phys Med Biol.

[44] Duck, F.A. in Physical properties of tissue: a comprehensive reference book, Edn. illustrated 73–135 (Academic Press, 1990).

[45] Cobbold, R.S.C. Foundations of biomedical ultrasound. *Oxford University Press* (2007).

[46] Grondin, J., Payen, T., Wang, S. & Konofagou, E.E. Real-time Monitoring of High Intensity Focused Ultrasound (HIFU) Ablation of *In Vitro* Canine Livers Using Harmonic Motion Imaging for Focused Ultrasound (HMIFU). *J Vis Exp*, e53050 (2015). 10.3791/53050 PMC4643394 26556647

[47] Downs, M. E., Buch, A., Karakatsani, M. E., Konofagou, E. E. & Ferrera, V. P. Blood-Brain Barrier Opening in Behaving Non-Human Primates via Focused Ultrasound with Systemically Administered Microbubbles. *Sci Rep-Uk* **5** (2015). 10.1038/srep15076 PMC4620488 26496829

